# In Vitro Fertilisation and Systemic Lupus Erythematosus: Making The Correct Choice

**DOI:** 10.7759/cureus.60100

**Published:** 2024-05-11

**Authors:** Pankti U Tripathi, Meenal M Patvekar, Prashant Suryarao, Shambhavi S Ghotankar

**Affiliations:** 1 Obstetrics and Gynaecology, Dr. DY Patil Medical College, Hospital and Research Centre, Pune, IND; 2 Central Research Facility, Dr. DY Patil Medical College, Hospital and Research Centre, Pune, IND

**Keywords:** complications, pregnancy, assisted reproductive technique, anti phospholipid antibody, systemic lupus erythematosus

## Abstract

Systemic lupus erythematosus (SLE) is a chronic autoimmune disease that increases the risk of complications in pregnancy. SLE flares during pregnancy are attributed to higher levels of estrogen and other cytokines. A 34-year-old woman with SLE, who had undergone in vitro fertilization (IVF), was diagnosed with a lupus flare at 14 weeks of gestation. Prior to conception, it is essential to evaluate disease activity, major organ complications, hypercoagulability, and any other medical conditions that could affect pregnancy outcomes in women with SLE in order to prevent adverse results. Additionally, the presence of anti-phospholipid antibodies (APLA) should be ruled out before considering assisted reproductive techniques (ART). After conception, optimal and vigilant monitoring is essential. A multidisciplinary team and approach should be employed throughout pregnancy to manage disease progression and identify any further complications. In conclusion, effective counseling and clear explanations of risks and benefits are pivotal in empowering the patient to make an informed decision.

## Introduction

Systemic lupus erythematosus (SLE) is an enduring inflammatory autoimmune disease with a complex aetiology. It is marked by the presence of non-organ-specific autoantibodies and encompasses a diverse array of clinical and immunological manifestations that can impact multiple organ systems [1]. Pregnancy, in particular, may exacerbate the condition by increasing the risk of complications such as hypertension, nephritis, recurrent abortion, and unfavourable neonatal outcomes [2]. Its incidence is 1 in 900 women. Additionally, one-third of women undergo cesarean sections, 33% experience preterm births, and over 20% develop hypertensive complications in pregnancy and lupus flares. An increase in disease activity in one or more organ systems accompanied by new or worsening clinical manifestations or laboratory findings is referred to as a lupus flare. These flares place the patient at risk of end-organ damage and are associated with morbidity and mortality. Increased SLE flares in pregnancy are attributed to higher amounts of estrogen, prolactin, and T-helper cell 2 cytokines [3]. SLE contributes to maternal mortality of 325 per 100,000 pregnant women.

For a woman, pregnancy is a period of rapid changes in physiological, psychological, and sociological dimensions, and SLE adds a burden to this. Ensuring the safety of elective procedures like in vitro fertilization (IVF) in patients with SLE, particularly those with positive antiphospholipid antibodies (APLA), is a significant concern that necessitates well-coordinated multidisciplinary management with rheumatologists, obstetricians, reproductive endocrinologists, and infertility experts [4]. Women with infertility who are diagnosed with SLE and desire pregnancy via elective procedures like IVF face another challenge, as IVF may require hormonal manipulation throughout pregnancy, which raises the risk of SLE flares and thromboembolism [5].

Despite advances in SLE treatment, the majority of patients continue to experience flares and remissions. Flare is a significant risk factor for organ damage and a poor prognosis for SLE, and it also imposes a significant psychological burden on patients. Currently, the cause of an SLE flare is unknown, but infection is considered a possibility [6].

We report a case of a 34-year-old woman who presented with early-onset severe hypertension, thrombocytopenia, hypothyroidism, and a lupus flare at 14 weeks of gestation. She was a known case of SLE who had undergone IVF.

## Case presentation

A 34-year-old G2A1 was referred from a tertiary care center to our emergency department with a history of four months of amenorrhea, conceived by IVF. She complained of bilateral lower limb swelling for five days along with a history of raised blood pressure for four days. The swelling was insidious in onset and of the pitting type.

She was hospitalised in a private hospital and started on antihypertensive medication for her condition. She was a known case of systemic lupus erythematosus (SLE) with secondary antiphospholipid antibody (APLA) syndrome for five years and was on multitarget drug therapy for the same, which included tablet hydroxychloroquine 300mg once daily and tablet prednisolone 20mg once daily along with calcium supplements.

In her past obstetric performance, the patient had a history of a missed abortion at 12 weeks of gestation for which dilation and evacuation were done, after which she was started on a tablet of aspirin 75mg. She is a known case of hypothyroidism on a tablet of thyroxine sodium 25 micrograms. Additionally, she had a history of pulmonary tuberculosis and had been on anti-tubercular therapy for six months. On admission to our emergency department, she was afebrile, and her vitals were as follows: pulse rate of 100 bpm, blood pressure of 170/120 mmHg, SpO2 100% on room air. Her BMI was 30.3.

Her physical examination revealed bilateral pedal oedema, pitting in nature. Her systemic examination was unremarkable. Per abdomen was soft and non-tender, and local examination had not revealed any active bleeding. On per-vaginal examination, the uterus corresponded to 14-16 weeks' size and was relaxed.

She was shifted to the intensive care unit and was closely monitored for all vital parameters, input-output and laboratory parameters. Her laboratory parameters revealed a decreased platelet count of 92,000/µL, haemoglobin was 10 g/dl. Liver and kidney function tests were normal. Blood profile also revealed hypocomplementemia (C3 - 70mg/dl), and urinalysis showed raised levels of proteinuria (+2) and total protein after 24 hours (200mg/L), which suggested renal involvement (Table 1). Her obstetric scan revealed a single live intrauterine gestation of 14 weeks. Mean uterine artery PI was normal for gestational age. Renal artery Doppler and bilateral lower limb venous Doppler were performed and found to be normal.

**Table 1:** Investigations on admission dsDNA: double-stranded deoxyribonucleic acid, nRNP: ribonuclear protein, Sm: Smith, +: present, -: absent Values are represented as quantitative and qualitative data.

Investigations	On admission	Reference range
Haemoglobin	10	11.6-15.0g/dL
Total leucocytes count	7200	400-10,000/µL
Platelet count	92,000	1,50,000-4,10,000/µL
Urine protein	2	-
Total urine protein	5664	1-119mg/L
Urinary proteins in 24 hours	200	10-140mg/L
C3 complement	70	90-180mg/dL
C4 complement	9.1	10-40mg/dL
dsDNA antigen	+	-
nRNP/Sm antigen	+	-
Nucleosome antigen	+	-
Histone antigen	+	-
Ribosomal P protein antigen	+	-

In view of the above clinical findings, a provisional diagnosis of a lupus flare with lupus nephritis was made as the patient had new-onset proteinuria, hypocomplementemia, thrombocytopenia, along with hypertension.

The patient was immediately started on glucocorticoid therapy (methylprednisolone 250mg injection). Labetalol injections were administered followed by continuous labetalol infusions for the control of hypertension.

A multidisciplinary approach with the rheumatologist, nephrologist, and intensivist was sought in the management of this patient, and considering severe blood pressure, proteinuria and deranged blood parameters, a call for a therapeutic abortion was made. The patient and the relatives were thoroughly counselled, and the risks and complications of continuing the pregnancy and delaying the treatment were explained in depth. The patient consented to the termination of pregnancy.

A team was prepared, and after taking the high-risk consents, the termination procedure was done with the use of tablet misoprostol 200mg every four hours. The induction abortion interval was 12 hours.

Her post-abortion course was uneventful, and nephrology and rheumatology lab tests suggested no abnormalities.

Over the next week, the blood pressure settled down, protein was reduced to trace amounts in the urine, and platelet count gradually increased to 1,25,000/µL after which the patient was discharged and asked to follow up after six weeks with the nephrologist for a renal biopsy.

## Discussion

Women with SLE may encounter infertility and decide to try artificial reproductive techniques (ART). ART increases the risk of thrombosis, flares and pregnancy-related problems because it is a hormonally induced process.

Patients diagnosed with systemic lupus erythematosus (SLE) often face a challenging outlook when it comes to pregnancy due to the significant risks of maternal and fetal complications. These include spontaneous miscarriage, preeclampsia, intrauterine growth restriction, fetal mortality, preterm birth, and ovarian hyperstimulation [2,3,4]. 

In the past few years, the outcomes of pregnancy in patients with SLE have been enhanced through various measures. These include preconception counselling, careful monitoring throughout pregnancy, and comprehensive management involving a multidisciplinary approach [7]. Nevertheless, a recent meta-analysis conducted to compare the maternal and fetal outcomes between women with and without systemic lupus erythematosus (SLE) revealed that pregnancies of women with SLE still exhibit a higher prevalence of adverse outcomes. These outcomes include spontaneous abortion (relative risk [RR] of 1.51), preeclampsia (RR of 1.91), thromboembolic disease (RR of 11.29), and preterm birth (RR of 3.05) [8]. Moreover, it has been estimated that women diagnosed with SLE experience a lower number of live births compared to the overall population [9].

In this case report the patient was diagnosed to have SLE and secondary APLA along with other comorbidities even before she embarked on her pregnancy and later developed early-onset hypertension involving renal and haematological manifestations. Therefore it led to a lot of problems after conception and the pregnancy was terminated. These observations support that IVF-associated pregnancy with SLE can lead to poor pregnancy outcomes.

**Figure 1:**
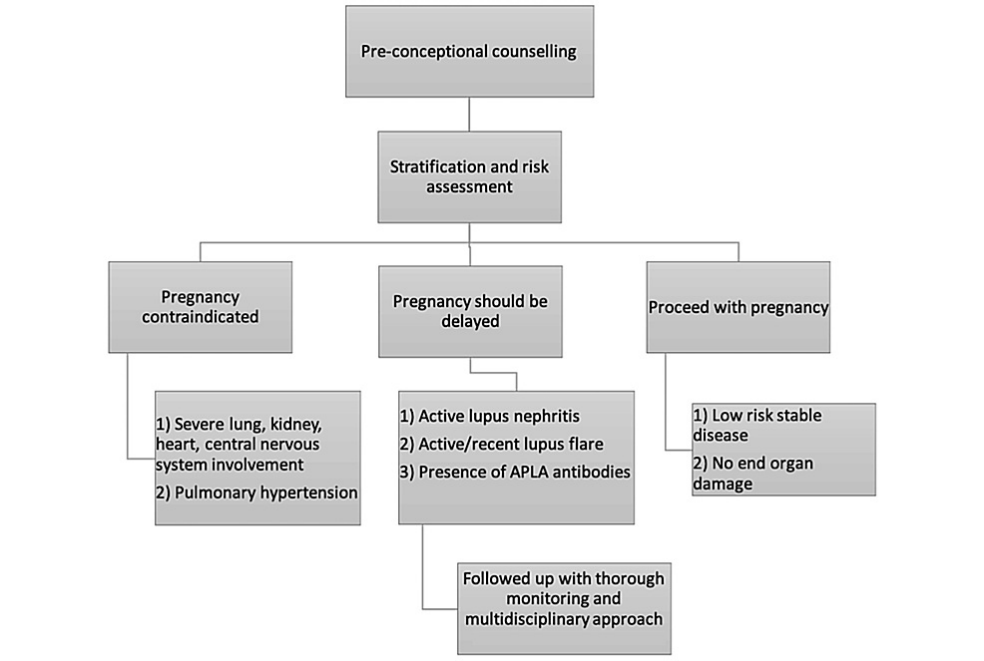
Stratification and risk assessment before pregnancy Credit: Pankti Tripathi, Meenal Patvekar

It is important to determine each patient's risk of experiencing medical issues during treatment and pregnancy before they seek fertility services. Though IVF is dangerous in SLE, good preconception counselling and quiescent disease activity for at least six months along with the absence of APLA and renal involvement is necessary for a better outcome [1]. Also, patients with SLE undergoing such procedures should be supervised and monitored efficiently throughout pregnancy (Figure 1). 

**Figure 2:**
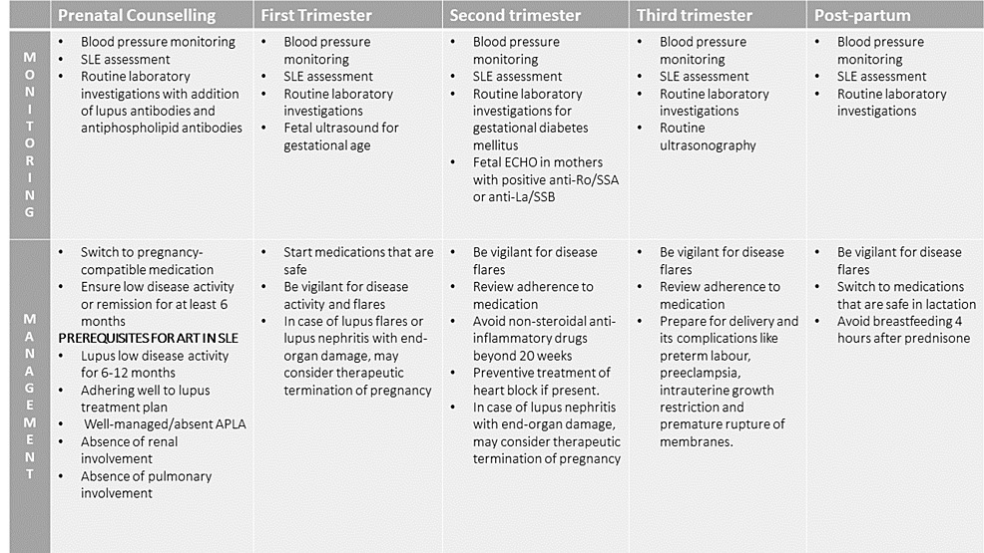
Management and planning of pregnancy in SLE Credit: Pankti Tripathi, Meenal Patvekar

Hence patient selection is of paramount importance. Women who are more likely to experience difficulties during fertility therapy or pregnancy should get detailed counselling from clinicians about these concerns. Experts in feto-maternal medicine or those knowledgeable about the woman's specific medical situation should be included in this counselling. Risks to the woman's fertility, her pregnancy, and the unborn child should all be discussed in counselling. Before deciding whether to start therapy or not, such counselling should take place. Clinicians may also decide to deny therapy if they believe that the patient's medical risks are too great for them to treat them ethically, based on unbiased and evidence-based assessments. Counselling plays a crucial role in ensuring that the patient and her family have a thorough understanding of the diagnosis, treatment options, and potential risks and benefits (Figure 2).

## Conclusions

A woman’s health status before conception is important for a successful outcome. Hence, in women with SLE, patient selection and pre-conceptional counselling and assessment are essential to determine whether pregnancy may pose high maternal or foetal risks. Medical management of such patients has to be weighed in a way that the benefit is more than the risk. Prior to pregnancy, it is crucial to conduct thorough investigations in women with SLE. These investigations should encompass an evaluation of disease activity and the extent of major organ involvement. Additionally, it is important to assess hypercoagulability or any concurrent medical disorders that could potentially affect the pregnancy. By conducting these investigations, healthcare professionals can effectively identify any potential risks and take appropriate measures to prevent unfavourable outcomes. The presence of APLA should also be ruled out before considering ART. After conception, optimal and vigilant monitoring is essential. A multidisciplinary team and approach should be employed throughout pregnancy to take control of the disease and its progression and maintain a vigilant eye to identify and manage complications if they arise. In summary, effective counselling and risk-benefit explanations are pivotal in empowering the patient to make an informed decision.
